# Untangling the role of environmental and host-related determinants for on-farm transmission of verotoxin-producing *Escherichia coli* O157

**DOI:** 10.1080/20008686.2024.2406852

**Published:** 2024-10-08

**Authors:** Lena-Mari Tamminen, Johan Dicksved, Erik Eriksson, Linda J. Keeling, Ulf Emanuelson

**Affiliations:** a Department of Clinical Sciences, Swedish University of Agricultural Sciences, Uppsala, Sweden; b Department of Applied Animal Science and Welfare, Swedish University of Agricultural Sciences, Uppsala, Sweden; c Swedish Veterinary Agency (SVA), Uppsala, Sweden

**Keywords:** VTEC O157, cattle, EHEC, epidemiology, super-shedder

## Abstract

**Background:** Cattle colonised by the zoonotic pathogen verotoxin-producing Escherichia coli of serotype O157 (VTEC O157) can shed high levels of the pathogen in their faeces. A suggested key for controlling VTEC O157 is preventing colonisation of individuals.

**Aim:** In this study the role of individual super-shedders and factors related to susceptibility and environmental exposure in the transmission of VTEC O157 among dairy calves are explored.

**Methods:** The association between sex, age, pen hygiene, pen type and stocking density and colonisation of individual calves, established by recto-anal mucosal swabs, on farms where pathogenic VTEC O157 had been confirmed was investigated. In a follow-up sampling, the consistency of previously identified risk factors and the role of shedding pen mates was assessed by studying the risk of new/re-colonisation.

**Results:** The results suggest an important role of stocking density that decreases with age, possibly due to increased resistance to colonisation following exposure. However, previous colonisation did not influence the risk of being colonised in the second sampling. Super-shedders (shedding >103 colony forming units/g faeces) significantly increased the risk of colonisation in peers (OR = 10, CI 4.2–52). In addition, environmental factors associated with survival of the bacteria, affected risk.

**Conclusion:** The results confirm the suggested importance of super-shedders but also emphasises the importance of considering the combined exposure from peers and the environment.

## Introduction

Since the first reported outbreak in 1982, verotoxin producing *Escherichia coli* (VTEC) has become recognised as an important cause of gastrointestinal disease in humans worldwide [1,2]. The serotype O157 is considered one of the most pathogenic due to its high prevalence among outbreak VTEC and its association with the severe complication haemolytic uremic syndrome (HUS) [3]. The main reservoir of VTEC O157 is cattle and transmission can occur by direct contact with animals or an environment in which colonised animals are shedding the bacterium, as well as through food contaminated by VTEC O157 [4–6].

Although VTEC O157 has high potential for causing serious disease in humans, it causes relatively mild local inflammation in cattle [7,8]. Colonisation of the recto-anal junction (in the terminal rectum) is associated with increased shedding levels, so called super-shedding, which has been suggested to have an important role in the epidemiology of VTEC O157 [9,10]. Super-shedding has been loosely defined as shedding more than 10^4^ colony forming units (cfu) per gram faeces but shedding more than 10^3^ has recently been proposed as an alternative definition [9,11]. Super-shedding cattle have been observed to contribute more than 90% of VTEC O157 shed on farms, though they constitute a small proportion (~10%) of the total cattle [12,13]. It has been proposed that super-shedding animals increase the risk of dissemination and persistence of VTEC O157 on farms [9,11,14,15]. While the importance of super-shedding is well recognised, longitudinal studies suggest substantial within- and between-host variability in colonisation and shedding over time [16–18]. The variability between individuals suggests that it is not an individual subgroup of animals that remain colonised over time that are solely responsible for super-shedding but the heterogeneity in individual shedding makes it difficult to fully evaluate the importance of specific individuals.

Transmission and exposure to VTEC O157 is not only driven by shedding animals. Environmental factors related to exposure, like faecal contamination of bedding and dryness of bedding, have been observed to have an impact [19–21]. As a result, cleaning and hygiene measures that target environmental growth and maintenance, such as keeping bedding dry and applying slaked lime, have been proposed to reduce the number of animals colonised by VTEC O157 on farms [22–24]. However, hygiene measures have little impact on already colonised individuals and do not reduce exposure to shedding peers.

In addition to environmental factors, host related variables appear to influence the risk of colonisation. Calves and young stock are considered to be more likely to be colonised compared to older animals and 2 to 6 months of age is often pointed out as a risk period. However, others have suggested an increased susceptibility between 1–3 months and up to 12 months [25–28]. In addition, weaning has been observed to be associated with an increased risk of colonisation, possibly due to the change in diet [12,29,30]. However, a link between colonisation and individual differences in behaviour, such as frequency of self-grooming and other behaviours possibly related to increased pathogen exposure, has also been suggested [31].

Thus, it is clear that transmission of VTEC O157 is multifactorial and involves both host and environmental factors that are closely connected. For example, weaning and young age are not only associated with changes in diet, but also with changes in management and housing as well as social contacts. The aim of this study was to investigate the effects of previously suggested risk factors (presence of super-shedders, type of pen, other animals in pen, environmental contamination and age) on transmission and individual colonisation from a multifactorial perspective that includes confounding, effect modification and interactions. We establish that all included dairy calves are exposed to VTEC O157 and evaluate the repeatability of the environmental risk factors as well as the role of previous colonisation status on the risk of becoming colonised during a follow-up period of 5 weeks.

## Materials and methods

### Sampling population and study design

The study was performed on 12 Swedish dairy farms where presence of VTEC O157 had been established by environmental sampling consisting of pooled pat samples (fresh manure from 15 to 20 pick-points in the pen) and over-shoe samples (walking around the pen with a gauze soaked with phosphate buffer fitted over plastic overshoes) from calves (<6 months) and young stock (6–12 months) [28,32]. After confirmation of a positive farm a more thorough sampling to identify positive groups of animals on farm was performed. Over-shoe samples from pens of non-weaned calves (~0–2 months), weaned calves (2–6 months), young stock (6–12 months) were collected [31]. If the described groups were larger than 50 individuals housed in multiple pens or included animals housed in separate buildings additional environmental samples were collected.

**Figure 1.**
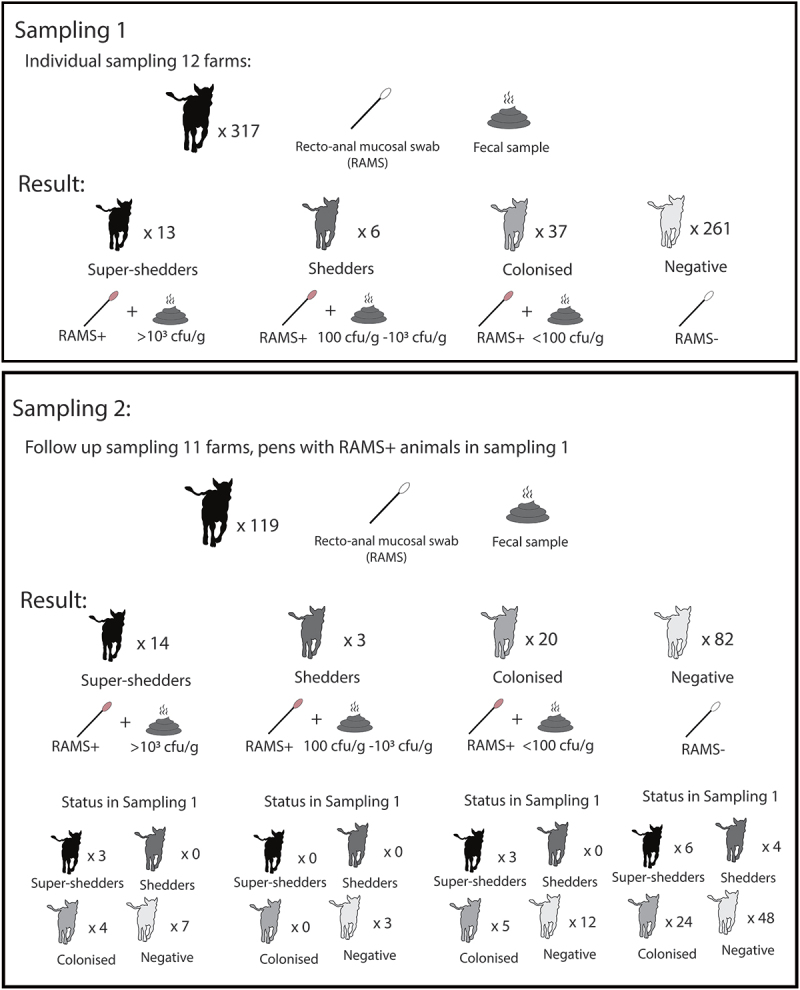
Schematic overview of results from first and second sampling. (cfu/g = colony forming units of VTEC 0157 per gram faeces)

Within 2 weeks of the thorough sampling the farms were revisited and animals from pens indicated as positive for VTEC O157 by environmental sampling were selected for individual sampling [31]. In total, 317 group-housed calves between 7 and 302 days of age from 52 pens where VTEC 0157 was present were sampled (Figure 1). Between 18 and 29 animals were sampled on each farm. The aim was to sample a minimum of 20 individuals on each farm (provided that number of individuals was present in the positive pens) but this number was increased when practically possible. When the total number of animals in pens with VTEC O157 was more than 20, a subset of individuals were selected for sampling either by simple random sampling (writing the numbers on notes and randomly drawing the number to be sampled) or systematic randomisation. Systematic randomisation was performed in large pens. The calf closest was first selected followed by every second or every third calf by distance from the sampler. The number of animals sampled per farm and pen is presented in Table 1. Approximately 5 weeks after the first visit, 11 of the farms were revisited (one farm had let animals out on pasture where they could not be restrained for sampling). A previous study has showed that monthly sampling detected changes in colonisation status [18]. During the second visit previously colonised animals were resampled. In addition, for each colonised individual 2–3 previously negative animals housed with the colonised animal were randomly selected and resampled. In total 119 individuals were included in the second sampling (Figure 1).

**Table 1.** Description of pens where environmental sampling indicated presence of verotoxin producing *Escherichia coli* (VTEC) O157 and the results from the individual sampling (pens with animals shedding high levels, like >10^3^ or >10^4^cfu/g, are included in the total number of pens with animal/s shedding >0 cfu/g).

					Number of pens with			
Farm	Pens where calves were sampled	Number of animals in pens	Animals sampled in pens (%)	Range of average calf age (days) in sampled pens	Colonised animal/s	Shedding animal/s (>0 cfu/g)	Super-shedding animal/s (>10^3^cfu/g)	Super-shedding animal/s (>10^4^cfu/g)
F1	5	5–8	3–6 (50−100%)	94–227	2	1	1	1
F2	4	10–12	5–8 (42−73%)	75–196	2	1	0	0
F3	4	9–21	1–10 (10−48%)	85–155	1	1	1	1
F4	2	7–13	7–13 (100%)	111–158	1	1	1	1
F5	4	7–14	5–9 (63−71%)	82–154	1	1	1	1
F6	5	4–20	3–14 (70−75%)	33–99	2	1	0	0
F7	7	4–17	3–7 (18−100%)	12–127	4	3	3	3
F8	4	7–18	4-8 (44−67%)	46–219	3	1	1	1
F9	7	6–36	5-14 (39−100%)	76–176	4	4	3	2
F10	3	6–13	6-12 (54−100%)	61–195	2	1	1	1
F11	6	2–6	(2-6) (67−100%)	12–171	3	0	0	0
F12	1	19	18 (95%)	216	1	1	0	0

All sampling was performed by the first author according to the ethical approval (Uppsala Djurförsöksetiska Nämnd, Dnr: C 85/15). Calves were restrained individually or in groups. First a faecal sample was collected directly from the rectum by rectal stimulation and placed in a plastic jar. After this, a recto-anal mucosal swab (RAMS) was collected using a foam coated cotton swab which was rubbed against the rectoanal junction (approximately 2–5 cm from the terminal rectum) for 1 minute. The swab was placed into a 15 ml sterile tube containing 2.5 ml phosphate buffer. Samples were sent to the Swedish veterinary institute (SVA) in Uppsala and analysis started within two days after sampling.

### Microbiological analysis

For each environmental sample (the pair of gauzes or 25 mg of feces), 225 ml of modified tryptic soy broth (mTSB) (Oxoid) (supplemented with 20 mg/l of novobiocin) was added and mixed with the sample in a stomacher.

Samples were then pre-enriched at 41.5°C ±0.5°C for 18–24 h. Following enrichment (41.5°C ±0.5°C for 18–24 h), immunomagnetic separation (using Dynabeads anti-*E. coli* O157; Thermo Fisher) was performed. Beads were spread on sorbitol McConkey agar (Oxoid) supplemented with potassium tellurite (2.5 mg/l) and cefixime (0.05 mg/l) (Dynal) (CT SMAC). Plates were incubated (37°C for 18–24 h) and up to 5 colonies with phenotype characteristic for serotype O157 (sorbitol negative) were tested by agglutination (latex kit DR 622; Oxoid).

Analysis of RAMS started immediately upon receipt by the laboratory, while faecal samples were stored at 2°C. Tubes with RAMS were vortexed and filled with 20 ml of tryptic soy broth supplemented with novobiocin (20 mg/l) before enrichment, immunomagnetic separation, culture on CT SMAC agar as described above. Calves were considered colonised if VTEC O157 was confirmed by agglutination of at least one colony with phenotypic characteristics (up to 5 colonies tested). Isolates from two positive animals per farm were further analysed for presence of genes encoding O157 (*rfbO157*), verotoxin 1 (*vtx1*) and verotoxin 2 (*vtx2*) as well as intimin (*eaeA*) [33,34]. The two isolates from each farm were also evaluated for belonging to clade 8 (a lineage associated with more severe disease in humans) by real time PCR [35](Riordan 2008).

VTEC O157 shedding levels of calves that were RAMS positive for VTEC O157 were determined by dissolving 10 g of the faecal sample in 90 ml of peptone salt solution and creating a series of 10-fold dilutions. From each dilution 0.1 ml was spread on CT SMAC agar. Concentration of bacteria per gram faeces was calculated based on the number of sorbitol negative colonies on the agar after 18–24 hours of incubation in 37°C. Calves with no sorbitol negative colonies (<100 cfu/g) were considered colonised but not shedding. A super-shedder was defined as an individual shedding >10^3^ cfu/g faeces (but we also present data on individuals shedding > 10^4^ cfu/g faeces). Animals shedding more than 100 cfu/g but less than 10^3^ cfu/g faeces are referred to as shedders.

### Risk factors evaluated

The variables evaluated in the study have been previously proposed to be risk factors for colonisation of cattle by VTEC O157 [19,22,23,30]. In this study, colonisation was defined as the presence of VTEC O157 in swabs of the terminal rectum. The variables analysed as potential risk factors in the first sampling and the follow-up sampling are presented in Tables 2 and 3. Distances for calculating pen area were measured using a laser telemeter. Pen cleanliness was scored by assessing visible faecal contamination in the bedding and scored as clean (Score 1: limited faeces visible, dry bedding), some dirt (Score 2: faecal contamination of bedding material clearly visible and/or bedding wet in part of the pen) or very dirty (Score 3: faecal contamination visible and/or bedding wet in the whole pen). Water/cattle ratio was calculated by dividing the number of water points with the number of animals in the pen. To create a stocking density measure that reflected the increase in weight as animals got older, an estimate for kilograms per square meter was calculated. The average age (in days) of calves within the pen was multiplied with the total number of calves in pen and average daily gain (estimated to be 0.81 kg) to create an estimate of kilograms within pen. This number was then divided by area of pen (m2). Pens were classified by type (thin bedding of straw/sawdust, slatted floor, concrete/rubber floor or deep straw bedding).

**Table 2.** Comparison of calves colonised with verotoxin producing *Escherichia coli* O157 and negative calves from a cross-sectional sampling on 12 Swedish dairy farms. Pen was included to account for random effects. 95% CI = 95% confidence intervals calculated using parametric bootstrapping (500 runs).

	Colonisation of VTEC O157*		Bivariate analysis	Multivariate analysis			
	No	Yes	p	OR	95% CI		p^§^
Number of calves	261	56					
Male calf (%)	90 (34.5)	9 (16.1)	0.007^†^	0.36	0.11–0.94		0.01
Age (months) (median [IQR])	4 [2.8, 5.4]	3.4 [2.6, 4.6]	0.11^¶^	1.24	0.80–2.23		0.30
Stocking density (10 kg/m^2^) (median [IQR])	2.46 [2.13, 3.34]	3.34 [2.45, 3.49]	0.002^¶^	1.99	1.12–4.47		0.01
Animals in pen (median [IQR])	11.00 [6.00, 14.00]	8.00 [6.00, 18.25]	0.686^¶^	1.02	0.91–1.13		0.62
Pentype (%)			0.15^†^				0.87
Straw/sawdust bedding	118 (45.2)	35 (62.5)					
Deepstraw bedding	114 (43.7)	18 (32.1)		0.64	0.23–2.17		
Concrete/Rubber	13 (5.0)	1 (1.8)		0.44	<0.01–23.2		
Slatted floor	16 (6.1)	2 (3.6)		0.68	<0.01–38.1		
Pen hygiene (%)			0.28^†^				0.57
Dry and clean	112 (42.9)	18 (32.1)					
Some dirt and partly wet	50 (19.2)	11 (19.6)		1.82	0.56–5.37		
Wet and dirty	99 (37.9)	27 (48.2)		1.42	0.54–3.76		
Water points/cattle (median [IQR])	0.11 [0.08, 0.14]	0.14 [0.10, 0.25]	0.002^¶^	12.00	0.02–7298		0.42
Interaction: Stocking density:Age				0.72	0.72–0.98		0.02

*As determined by rectoanal swabs of the terminal rectum.

^§^Likelihood ratio test.

^†^Fishers exact test (groupwise comparisons for multilevel variables).

^¶^Wilcoxon rank sum test.

**Table 3.** Results of follow-up sampling of 119 individuals (previously colonised by verotoxin producing *Escherichia coli* (VTEC) O157 and 2-3 previously negative animals per colonised animal).

	Colonisation of VTEC O157*		Bivariate analysis
	No	Yes	p
Number of calves	82	37	
Previous colonisation of VTEC O157%)	34 (41.5)	15 (40.5)	1.00^†^
Male calf (%)	21 (25.6)	16 (43.2)	0.09^†^
Age in months (median [IQR])	4.3 [3.5, 5.4]	4.0 [3.4, 4.5]	0.14^¶^
Super-shedder (>10^3^cfu/gram faeces) in pen (%)	34 (41.5)	28 (75.7)	0.001^†^
Stocking density (10 kg/m^2^) (median [IQR])	3.8 [2.7, 6.25]	3.1 [2.8, 5.1]	0.90^¶^
Animals in pen (median [IQR])	11.50 [6.00, 20.00]	9.00 [7.00, 20.00]	0.42^¶^
Type of pen (%)			0.33^†^
Deepstraw bedding	38 (46.3)	11 (29.7)	
Concrete/rubber	3 (3.7)	1 (2.7)	
Slatted floor	4 (4.9)	2 (5.4)	
Straw/Sawdust	37 (45.1)	23 (62.2)	
Pen hygiene – Faecal contamination of bedding (%)			0.04^†^
(1) No faecal contamination	13 (15.9)	14 (37.8)	
(2) Part of bedding	47 (57.3)	16 (43.2)	
(3) The whole bedding	22 (26.8)	7 (18.9)	
Pen hygiene – Wet bedding (%)			1.00^†^
(4) Dry	42 (51.2)	19 (51.4)	
(5) Partly wet	28 (34.1)	13 (35.1)	
(6) Wet	12 (14.6)	5 (13.5)	
Waterpoints/cattle ratio (median [IQR])	0.14 [0.10,0.17]	0.14 [0.10,0.17]	0.64^¶^
Moved between samplings (%)	45 (54.9)	18 (48.6)	0.56^†^
Weaned between samplings (%)	12 (14.6)	8 (21.6)	0.43^†^

*As determined by rectoanal swabs of the terminal rectum.

^†^Fishers exact test (groupwise comparison for multilevel variables).

^¶^Wilcoxon rank sum test.

Variables associated with risk of colonisation at the second sampling (around 5 weeks after the first) (Table 3) were measured and assessed as described for part 1, except that wetness and faecal contamination of pen were now scored separately. In addition, presence of a super-shedder in the pen in the previous sampling, previous colonisation status and if the calf had moved or been weaned between samplings were considered in the analysis.

### Statistical analysis

Data was entered into Excel and all statistical analysis performed in R [36]. Descriptive statistics and bivariate analysis were generated using the packages tableone and dplyr [37,38]. Association between colonisation of VTEC O157 (RAMS+ or RAMS-) and categorical variables were investigated using Fisher exact test as some groups were small (<10). To investigate associations with numerical variables a nonparametric Wilcoxon rank sum test was used.

Multivariable analysis of variable associations with colonisation (RAMS+ or RAMS-) were analysed with generalized mixed models (glm) with a logit link [39] in two steps. First, variables associated with colonisation in the first sampling were analysed in one model. Secondly variables associated with colonisation in the follow-up sampling were investigated in a separate model. Significance of variables was assessed using likelihood ratio test. Due to a smaller sample size in combination with an increased number of predictors in the follow-up sampling, the second model was reduced using likelihood ratio test and variables that did not significantly improve model (*p* > 0.05) were removed. Following reduction all removed variables were reintroduced one by one and changes in estimates > 20% were noted to evaluate signs of confounding. The variables faecal contamination and moisture were collapsed to two levels by combining pens with score 2 or 3. This was done because retaining the levels introduced multicollinearity (VIF >4) to the model. Likelihood ratio test did not indicate that this affected the model nor did the predictive capabilities change. Stocking density was scaled (from kg/m^2^ to 10 kg/m^2^) so the range of values was more similar to other variables. Pen was included as a random effect to account for clustering in both models.

Variance inflation factors (VIF) were calculated using the package car and VIF > 4 considered to suggest problems with collinearity [40]. Plausible interactions were tested and included if they significantly improved the variability explained by the model (determined by likelihood ratio test). Non-linearity of associations (main effects and interactions) were investigated using generalized additive models [41]. Model performances were evaluated using area under the curve [42]. Plots visualising predicted probabilities of colonisation were generated using the packages ggplot and ggeffects [43,44].

## Results

### First sampling

Of the 317 individuals sampled in the first sampling, 56 were colonised by VTEC O157 (as established by positive RAMS) and 19 were shedding the pathogen (>100 cfu/g faeces) (Figure 1). Out of these, 13 were super shedders (>10^3^ cfu/gram faeces). Colonised calves were found on all farms from 26 out of 52 sampled pens. Out of isolates subjected to further analysis (2 per farm) all except 2 belonged to clade 8 and had the virulence genes *eae* and *vtx2*. The 2 isolates from the remaining farm (F12) had the virulence genes *eae* and *vtx1* and did not belong to clade 8. This farm was also the most distant geographically from the other sampled farms as it was located in the south of Sweden while the others were located in an area of southeast Sweden. In addition, F12 could not be visited for follow-up sampling as animals had been let out to pasture.

In Table 1, the pens sampled on each farms and the number of pens where colonised and shedding animals were identified are presented. The results from the risk factor analysis are presented in Table 2 and suggest that male calves are less likely to be colonised by VTEC O157 (p < 0.01). In addition, stocking density was significantly associated with colonisation but the effect was influenced by an interaction with age. The interaction significantly improved variability explained by the model as determined by likelihood ratio test (p = 0.02).

**Figure 2.**
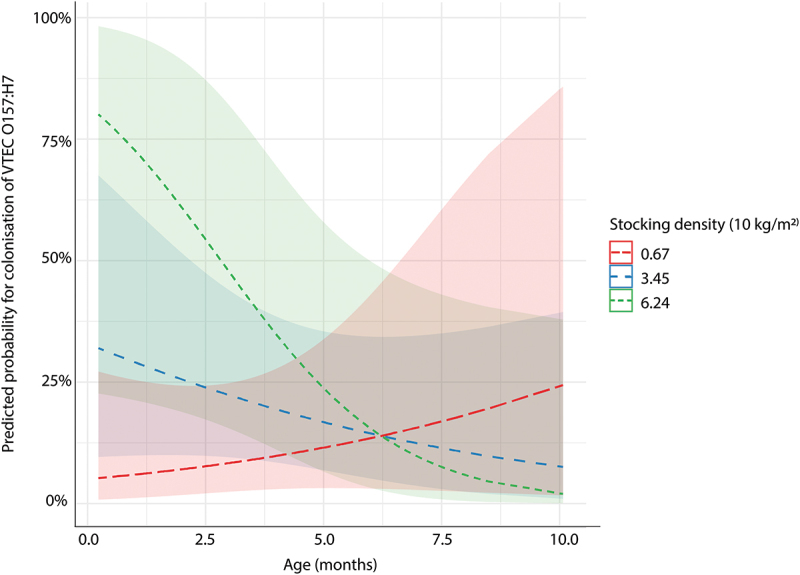
Illustration of the interaction between calf age (months) and stocking density on the predicted probability of colonisation by verotoxin producing *Escherichia coli* O157 (shaded areas are 95% confidence intervals). Effect of stocking density plotted for mean stocking density (34.5 kg/m^2^) and one standard deviation above and below mean (6.7 kg/m^2^, 62.7 kg/m^2^).

As illustrated in Figure 2, the risk for colonisation decreased with age for animals kept in high stocking density while it increased with age in groups of animals kept in low stocking density (*p* = 0.02). However, the confidence intervals are large for young animals housed in high stocking density and for animals in low density after 5 months of age. There were no indications of non-linear associations but removing variables led to changes in estimates which indicated confounding between sex and several variables (e.g. age, number of animals, water related variables, pen hygiene). This may reflect differences between male and female calves in our study population, for example male calves were younger and kept in cleaner pens, or indicate that there were uncontrolled confounders. The estimated variance of the random effect pen was 0.68 (standard deviation = 0.82), giving an intra class correlation of 0.17. This means that within pen variation is relatively small and that between pen variance (uncontrolled pen level variables influencing colonisation) are influencing remaining deviation. VIF was below 4 for all variables except pentype (VIF = 11). Rerunning the analysis without this variable led to small changes (<20%) in all estimates except the estimate for waterpoints/cattle which changed to OR 34, indicating a connection between pentype and water availability. However, this did not change the interpretation of the model or the predictive capabilities of the model as area under the curve, indicating the ability of the model to classify between RAMS+ and RAMS- calves, was 85% for both models (with and without pentype).

### Follow-up sampling

**Figure 3.**
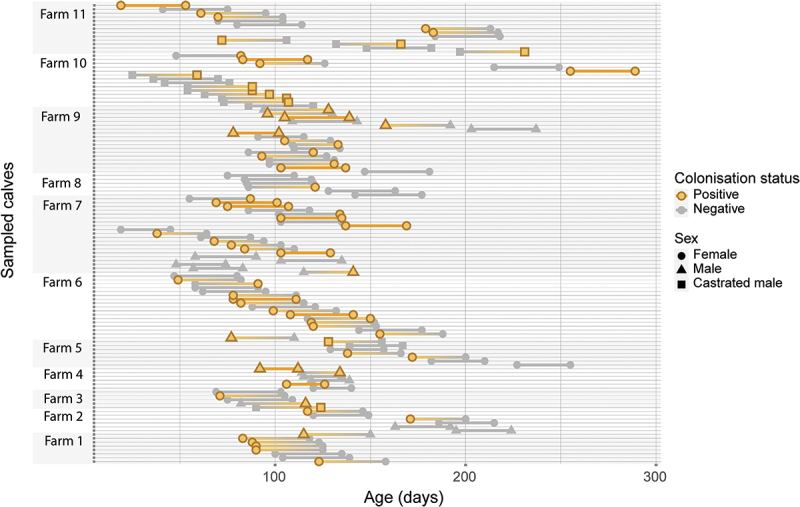
Colonisation status of verotoxin producing *Escherichia coli* O157 as determined by recto-anal mucosal swabs from calves sampled on two occasions with approximately 5 weeks between samplings. Each point represents a sampling occasion, colour status on sampling and shape indicates sex of the calf.

In the follow-up sampling, 119 calves from pens with RAMS+ animals were sampled. An overview of animals sampled in the first and second sampling, including previous status is presented in Figure 1. Of the 119 sampled animals, 37 animals (31%) were colonised by VTEC O157 (i.e. RAMS+). Of the colonised animals 13 were super-shedding (>10^3^ cfu/g) and 4 were shedding lower levels. Of these, 15 individuals from 5 farms remained colonised and the others had cleared infection. Three individuals were super-shedding on both sampling occasions. Twenty-two (31%) of the previously negative animals had become colonised. No apparent pattern between calf age, sex, farm and status in the two samplings was observed (Figure 3).

**Figure 4:**
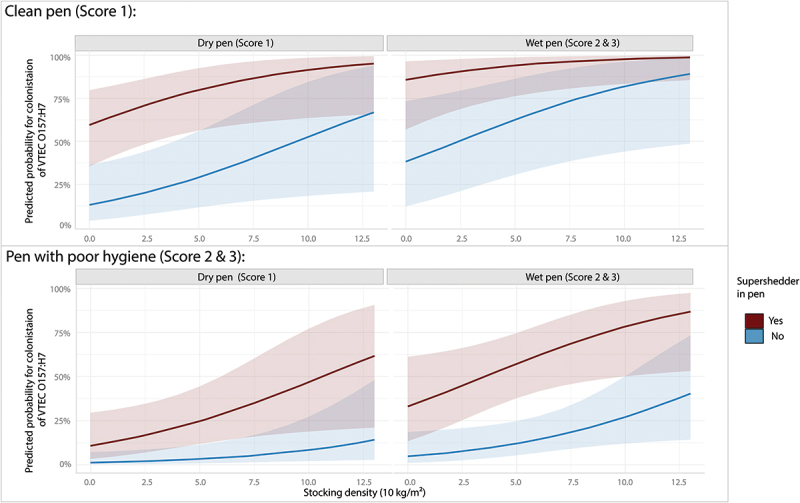
Proportional distribution of pen hygiene scores (faecal contamination/wetness of bedding).

**Figure 5.**
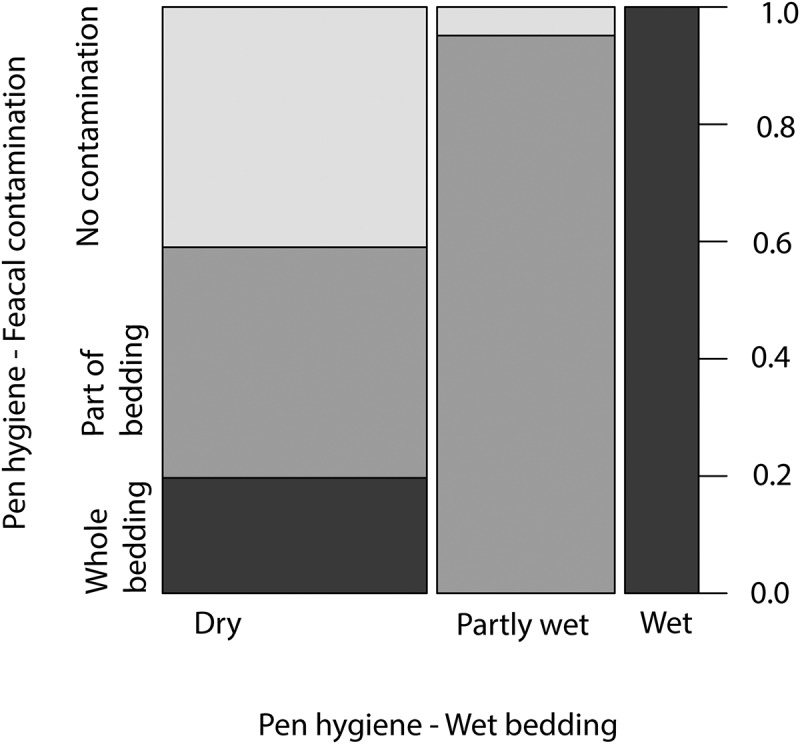
Predicted probabilities for colonisation of verotoxin producing *Escherichia coli* O157 in the follow-up sampling 5 weeks after the first sampling. Shaded areas are 95% confidence intervals.

Descriptive characteristics and analysis of the association between investigated variables and colonisation of VTEC O157 at the second sampling are presented in Table 3. In contrast to the first sampling, this sampling did not show a significant difference between sex. The colonised animals were on average slightly younger, although the difference was not significant, and a significantly larger proportion were kept in pens with good cleanliness (*p* = 0.04) and had been housed with a super shedder on the first sampling (*p* < 0.001). The final multivariable model included the presence of a shedder in pen during the previous sampling, pen hygiene, wetness of pen and stocking density (Table 4). Pen was included to control for clustering but did not explain any variance. There were no indications of non-linear associations and reintroducing any removed variable to the model did not influence estimates more than 20%. VIF for all variables was below 2 after faecal contamination of pen and wetness of pen had been condensed by combining score 2 and 3 which showed signs of correlations in the higher scores (Figure4) Predicted probabilities for colonisation in the final model are visualised in Figure 5. Increasing stocking density, being housed in a wet pen and being housed with a super shedder increased risk of being colonised in the second sampling. However, being kept in a dirty pen (with faecal contamination of bedding material) decreased risk of colonisation.

**Table 4.** Estimates and odds ratios of risk factors for being colonised by verotoxin producing *Escherichia coli* (VTEC) O157 in the follow-up sampling. Pen was included as a random effect (variance explained = 0). 95% CI = 95% confidence intervals calculated using parametric bootstrapping (500 runs). Area under the curve = 0.794.

Final model		95% CI		
	OR	Lower	Upper	p*
Super-shedder (>10^3^cfu/gram faeces) in pen	9.73	4.16	52.33	<<0.01
Pen hygiene – Faecal contamination of bedding (Score 2 or 3)	0.08	0.01	0.27	<<0.01
Stocking density (10 kg/m^2^)	1.21	0.96	1.55	0.04
Pen hygiene – Wet bedding (Score 2 or 3)	4.01	1.41	19.02	0.01

Note: *Likelihood ratio test.

## Discussion

The target of this study was calves in pens where virulent VTEC O157 was present. From all farms except one isolates of the hyper virulent type clade 8 were confirmed. Clade 8 is a virulent subgroup within serotype O157:H7 which is associated with more severe disease and a higher frequency of HUS [45]. In Sweden cases of VTEC O157:H7 are increasing and especially worrying is the high number of cases and outbreaks caused by clade 8 [46]. This is not surprising as all remaining farms were located in an area where the presence and circulation of closely related strains of VTECO157 has been previously reported [32]. The remaining farm from the south of Sweden (F12) had been associated with a case of human disease but the isolates showed a different virulence profile as they did not belong to clade 8 and had genes coding for vtx1 and not vtx2. A difference between strains of VTEC O157 may also mean differences in colonisation and shedding as well as survival in the environment [46]. Thus, while the follow-up sampling on this farm was not performed due to practical reasons, the exclusion of it in the follow-up study may have reduced the effects of variability between strains in the second part of the analysis.

By using environmental sampling to identify positive farms and groups of animals we were able to increase the likelihood of identifying colonised animals, which is often a problem in studies of this pathogen. The approach also made us more certain that animals in the study have had the opportunity to be exposed. Although presence of the pathogen is only part of the infectious process it is a requirement (‘necessary cause’) and by avoiding unexposed animals we reduced noise that would be introduced by including pens where the pathogen was not present. However, the targeted approach means that the proportions of positive animals should not be seen as estimates of on farm prevalence. The results confirm the importance of super-shedders but also reveal an interplay of management related factors that can guide how measures for controlling transmission of the pathogen could be applied on infected farms.

Both parts of the study (sampling 1 and 2) suggest an important role of stocking density. High stocking density has been previously associated with increased risk of colonisation in field studies and identified as an important driver of disease in simulation models [11,19,47,48,49]. Interestingly the results of this study suggests a more complex association between stocking density and age. Results from the first sampling suggest that risk of colonisation decreases with increasing age in groups of animals housed in high stocking density while it increases with age in animals housed in low stocking density. The pattern could represent a faster transmission, and immunity, developing in high stocking densities, while slower transmission in pens with lower stocking density leads to colonisation at a later age and that immunity in the group develops slower. There are several reasons to propose that immunity may be involved. Firstly, immunity to VTEC O157 has been studied in efforts to develop a vaccine against colonisation and the results indicate that immunisation can at least partly reduce shedding levels (reviewed by [50]. Secondly, calves inoculated multiple times with a strain of VTEC O157 have also been shown to shed for shorter duration [51]. It is likely that natural infection can also activate the immune response and that cattle develop immunity with age, which would explain why VTEC O157 colonisation is more prevalent among younger animals compared to adult cattle [25,29]. Thirdly, the finding that animals housed in pens with faecal contamination of bedding material were less likely to be colonised in the second sampling may also be associated with immunity following previous exposure. However, one major finding contradicts this reasoning. There were no indications that previously colonised individuals were less likely to be colonised, which would have been expected if previous colonisation led to immunity. This suggests that immunity may be relatively weak, require multiple infections or that there are other explanations. There are also other possible reasons for decreased susceptibility with increasing age. For example, diversity of the microbiota has been associated with increasing age and reduced risk of colonisation [25]. Behaviour and social contacts may also shift as animals get older and influence transmission, which could explain that a larger proportion animals are positive early in life.

Other members of the microbial community are not only important in the intestine but may also influence survival of VTEC O157 in the environment. Faecal contamination of bedding may lead to increased competition with other bacteria more adapted to surviving outside cattle. It has been suggested that more diverse microbial community negatively impacts survival of VTEC O157 in the environment (reviewed by [52] which could explain the protective effect of manure contamination of the bedding. Moisture is another important factor shown to influence both growth of VTEC O157 directly and through its effect on other members of the microbiota in dairy compost [53]. Varying levels and proportions of moisture and manure has been observed to modulate regrowth potential of VTEC O157. For example low growth and viability has been observed both in dry bedding (0.11 g H_2_O/g surface material) with 5% manure as well as beddings with high water content (1.5 g H_2_O/g surface material) and 75% faecal contamination while thriving in beddings with 25% manure and less moisture (0.43 g H_2_O/g surface material) [54]. Although the underlying mechanisms require further study, our results from the second sampling confirm the importance of considering both moisture and contamination when studying transmission and persistence of VTEC O157. They also support the importance of providing dry bedding on farms infected with VTEC O157 to control the pathogen [23], especially when animals are housed in high stocking density. However, it should be noted that the study only assessed the pen hygiene at the two sampling occasions and this may not be representative for the time between samplings. Thus, these findings should be interpreted with caution.

The effect of a super shedder was highly significant and this effect was observed despite the risk of there being unaccounted shedders in some of the pens where all animals were not sampled (34 out of 52 pens). Unaccounted shedders (false negatives) increase the risk of type II error, i.e. the risk of not identifying a true difference between groups. Thus, the effect of super shedder may be underestimated in this study. Using the presence of a super-shedder instead of presence of shedder (>0 cfu/gram feces) significantly improved the model according to likelihood ratio test (*p* < 0.001) as well as AUC (from 75% to 79%). As super-shedding was related to the outcome (colonisation) in the first sampling this variable was not included in the first model. It is noteworthy that when presence of super-shedder was included no residual variation due to pen remained in the second model. These results confirm the important role of super-shedders and support that super shedding, or shedding events, highly influence transmission to other animals in the pen. However, this is an observational study and there may be unmeasured confounders on pen level related to development of super-shedders and transmission that are the underlying driver of the observed pattern.

Overall, the variables associated with increased risk of colonisation are related to pen characteristics and shedding of peers. In addition, no association between previous colonisation status and status in the follow-up sampling was observed. These findings support that super shedding and colonisation is not associated with a particular group of individuals but is a periodic state in different individuals. This suggests that control of VTEC O157 is most efficiently applied on group level measures and not on identifying particular individuals. While there were indications of change in susceptibility of calves with age and due to sex in the first cross sectional sampling these were not confirmed in the follow-up study when management related variables (pen hygiene, stocking density) and presence of super-shedder in pen was accounted for. However, it should be kept in mind that the second part of the study included fewer individuals and potentially lacked the power to detect associations of smaller magnitude. Similarly, very few animals were weaned in between samplings and the study most likely lacked the power to pick up associations between weaning and colonisation.

The results confirm the importance of animals shedding > 10^3^ cfu/g faeces and stocking density on transmission of VTEC O157 in dairy calves. They also suggest that the balance between moisture and faecal contamination, previously observed to influence growth of VTEC O157 in dairy compost bedding, is associated with risk of colonisation. No signs of previous colonisation status nor individual characteristics such as sex or age influencing the risk of colonisation after 5 weeks were observed. This suggests that the associations between colonisation and risk factors such as young age are mainly related to changes in management and exposure to the bacteria, not increased susceptibility to colonisation. This study emphasises the importance of considering the combined exposure from peers and environment for understanding the transmission and thus in designing control measures for VTEC O157.

## Data Availability

The dataset analysed during the study is available in the Mendeley Data repository, doi: 10.17632/dp4czxjwjp.1.
